# The effects of cultural engagement on health and well-being: a systematic review

**DOI:** 10.3389/fpubh.2024.1369066

**Published:** 2024-07-10

**Authors:** Erica Viola, Marco Martorana, Daniele Ceriotti, Marta De Vito, Damiano De Ambrosi, Fabrizio Faggiano

**Affiliations:** ^1^Department of Sustainable Development and Ecological Transition, University of Eastern Piedmont, Vercelli, Italy; ^2^Department of Statistics, Computer Science, and Applications “Giuseppe Parenti” (DiSIA), University of Florence, Florence, Tuscany, Italy; ^3^Department of Translational Medicine, University of Eastern Piedmont, Novara, Piedmont, Italy

**Keywords:** cultural engagement, leisure activities, health, well - being, quality of life

## Abstract

**Purpose:**

This paper examines the effectiveness of culture-based activities in improving health-related outcomes among middle-aged and older adults. Based on the biopsychosocial model, this review aims to explore the impact of cultural engagement on health and well-being.

**Methods:**

We conducted a systematic literature review based on peer-reviewed articles retrieved from various electronic databases. In total, 11 studies were included in this review. Our study population consisted of healthy adults aged over 40 years.

**Results:**

The results provide evidence of positive association between cultural participation and better mental health (e.g., cognitive decline, depression, anxiety), frailty, resilience, well-being and social relations.

**Conclusion:**

This review suggests that cultural engagement serves as an effective means for individuals to maintain and enhance their health and well-being. The field is mostly limited by the heterogeneity of the studies and poor conceptualization of cultural activities. Thus, it is recommended that future research consider the effects of different cultural interventions in developing effective strategies for promoting healthy lifestyles and enhancing quality of life in later stages of life.

## 1 Introduction

For many years, the concept of health has evolved from a mere absence of disease to a more comprehensive evaluation. In 1948, the World Health Organization (WHO) defined health as “a state of complete physical, mental, and social well-being and not merely the absence of disease or infirmity” (1). This marked the beginning of a process that shifted the concept of health from an individual perspective to a more social one (2). This evolution has culminated in the current vision of health, described as “the ability to adapt and self-manage” (3) (p. 2), emphasizing the development of personal capabilities.

Therefore, despite significant progress in disease treatment, in recent decades, many researchers have shifted their focus to exploring methods for enhancing and maintaining health and well-being, leveraging cognitive, emotional, and social resources to confront challenges and meet daily requirements effectively. In particular, artistic activities have received significant attention as a potential means to enhance the quality of life, especially among the older population (4, 5). This association is now widely recognized (6), emphasizing the significant role of culture as a determinant of individual psychological well-being (7–9), psychological flexibility and health (10). Evidence from a recent comprehensive scoping review highlights the beneficial outcomes of engaging in diverse cultural and arts events (4), relevant to both health promotion and prevention efforts by fostering health-promoting behaviors and aiding in illness prevention.

In light of the complex challenges of the aging population, understanding the role of culture in promoting health and well-being becomes increasingly important. By expanding and intensifying research in these areas, we can identify strategies to enhance quality of life in an economically advantageous, accessible, and enjoyable manner.

The aim of the present paper is to review current literature addressing the relationship between different forms of cultural engagement and health and well-being in people aged over 40 years. We chose to follow the biopsychosocial model as a comprehensive framework that considers the interconnected influence of biological, psychological, and social factors on human behavior and experiences. This approach allows for a nuanced analysis, fostering a deeper understanding of human functioning. Additionally, aligning with this model enhances the relevance and applicability of our research findings across various fields. In the context of this systematic review, we will distinguish between “receptive culture,” which encompasses visits to museums, galleries, art exhibitions, theaters, concerts, cultural festivals, and community events, and “cultural participation,” which refers to active engagement in one or more of these activities (4). Both types of activities involve aspects of artistic and cultural experience, ranging from creativity, cognitive and sensory stimulation, to social interaction (e.g., esthetic pleasure, and emotional evocation), which promote health (11, 12). However, differences emerge in the impact of receptive and participatory culture; moreover, studies show contrasting results. Although active cultural engagement interventions have shown greater benefits in terms of psychophysical outcomes (13, 14), other authors have found only the efficacy of receptive activities in supporting healthy aging, perhaps because they more consistently involve social interactions and movement, which are positively associated with healthy aging (11, 12). Further research is needed for a better understand the underlying reasons for such differences. There is still a lack of research that evaluates the overall impact of arts engagement on healthy aging in a comprehensive and integrated manner (11, 12).

Based on these observations, we address the following key questions:

- How might different forms of cultural engagement relate to health and well-being?
- What gaps exist in the current literature examining the effects of cultural engagement on health and well-being outcomes? Consequently, what further research is needed?
- What are the implications of the present literature for healthcare and cultural systems and policies?

## 2 Methods

### 2.1 Study design

This study can be classified as a systematic review.

### 2.2 Search strategy

**Figure 1:**
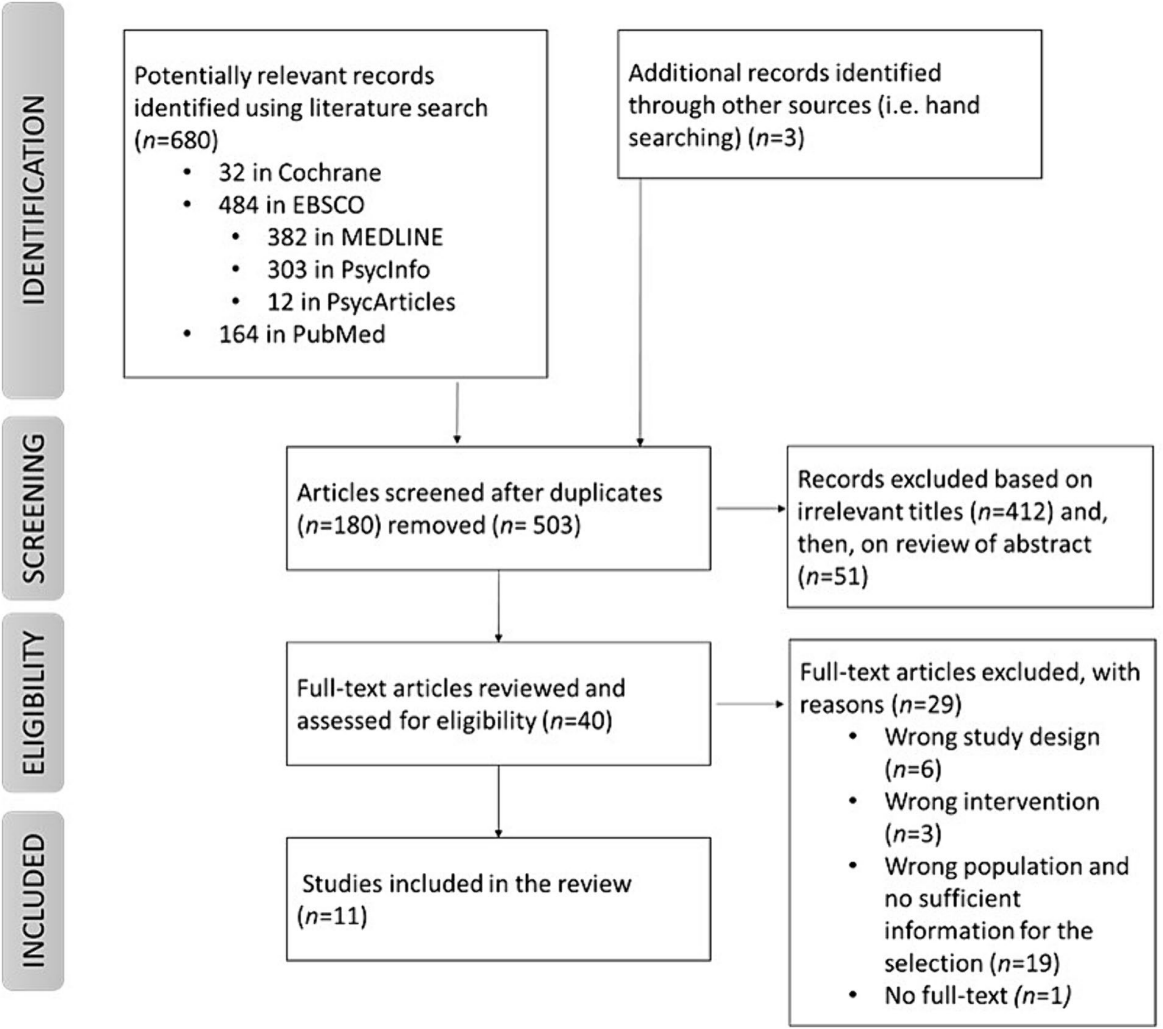
Flowchart of the literature identification and selection process.

A comprehensive search of published studies was conducted using the following databases: Cochrane, EBSCO and PubMed. Concerning the keywords, we considered very inclusive terms that refer to cultural engagement; regarding the effects, we have considered words related to health and well-being. The key terms for searches included: (“Cultural participation” OR “Cultural attendance” OR “cultural engagement” OR “cultural event*” OR “Art* activit*” OR “Art* participation” OR “Art* attendance”) AND (“Healthy lifestyle” OR “Health*” OR “health promotion” OR “Health behavior*” OR “well-being” OR “Well-being” OR “quality of life”). No publication date restriction was applied. Figure 1 presents the flowchart of the process of identifying and selecting literature. The selected articles were required to have undergone peer review processes prior to publication and to present a clear and consistent methodology. However, given the diverse methods and outcomes considered in the selected studies, this review will provide a qualitative synthesis of the results reported by the researchers.

### 2.3 Inclusion and exclusion criteria

Our criteria for inclusion were as follows: (1) quantitative methodology; (2) randomized controlled trial (RCT), longitudinal and cross-sectional studies with controls; (3) receptive arts engagement in terms of attendance of arts-based events such as museums, art exhibitions and galleries, concerts, the theater, and the cinema (15) as well as the active production of art (16); (4) according to the biopsychosocial approach, the consideration of physical, psychological and social variables associated to health and well-being as outcomes; (5) samples of healthy people aged over 40 years. The specific effects of music and/or making music on health were excluded in this study; instead, a separate study was dedicated to examining them (5). Systematic reviews and meta-analyses were also excluded.

### 2.4 Study selection

Our selection was conducted by screening articles titles, abstracts and considering full-text articles of potentially eligible papers. Three independent reviewers (EV, MM, DC) executed these procedures, resolving disagreements through discussion. The systematic review was undertaken according to the Preferred Reporting Items for Systematic Reviews and Meta-Analyses (PRISMA) guidelines (17).

### 2.5 Quality criteria

The methodological quality of the considered studies was analyzed using Checklist for Analytical Cross Sectional Studies (18). Two reviewers (EV and MM) assessed the methodological quality of included studies based on 8 criteria (see Supplementary Table A1). Each paper was assigned to be low (<5), moderate (between 5 and 7) or high quality (7 or 8) depending on the number of criteria they met; possible discrepancies were resolved by consensus. The results of the quality assessment process are listed in Supplementary Table A1.

## 3 Results

### 3.1 Search results

We identified 683 articles through the literature search process. After the exclusion of duplicates and following the inclusion/exclusion criteria, 11 studies were selected (see Figure 1). Summaries of the studies included in this review are presented in Table 1. All these studies examined the effects of cultural engagement on particular dimensions of health and well-being: mental health status, frailty, loneliness, and so forth. We present the results according to the specific outcome (Table 2 for effects and significance). In general, out of 95 overall effects, 42 statistically significant positive effects emerge (44%), whereas the remaining effects, although not statistically significant, are not negative and therefore do not worsen health and well-being. The most significant effects are derived from regular and sustained forms of cultural participation, whereas going to the cinema is found to be the least beneficial for health promotion.

**Table 1:** Detailed summary of the considered studies (alphabetical order).

First author, year	Country	N participants (% women)	Age (min-max)	Samples	Intervention / Data collection	Cultural variable classification	Outcome
Bolwerk, 2014 (16)	Germany	28 (53.5%)	62–70 (*M* = 63.7)	2 groups: art production group (*n* = 14) art evaluation group (*n* = 14)	RCT; weekly participation in two different 10-week-long art interventions (art production: active production of art in an art class; cognitive art evaluation: cognitive evaluation of artwork at a museum)	Production (active creation) or Evaluation (cognitive evaluation of artistic creations) of art in group interventions, 2 h and once a week for 10 weeks	Resilience
Fancourt, 2018 (19)	UK	3,911 (55%)	>50 (*M* = 63.8)	1 group (data from ELSA)	Longitudinal (data for a decade: 2004/2005-2014/2015) (Wave 2 – Wave 7)	Frequency of engagement in visiting museums, art galleries, and exhibitions on a six-point Likert scale (‘never’, ‘less than once a year’, ‘about once or twice a year’, ‘every few months’, ‘about once a month’, ‘twice a month or more’) merging the last 3 categories (‘every few months or more’) for a four-point scale	Diagnosis of Dementia
Fancourt, 2018 (20)	UK	3,445 (55.2%)	52–90 (*M* = 62.9)	1 group (data from ELSA)	Longitudinal (data for a decade: 2004/2005-2014/2015) (Wave 2 – Wave 7)	Frequency of engagement in visiting: (i) art gallery, museum or exhibition, (ii) theater, concert or opera (iii)cinema on a six-point Likert scale (from ‘never’ to ‘twice a month or more’) merging the last 2 categories for a five-point scale	Cognitive functions
Fancourt, 2019 (21)	UK	8,780	≥50	2 groups (data from ELSA): Infrequent cultural engagement; Frequent cultural engagement	Longitudinal (data for a decade: 2004/2005-2016/2017) (Wave 2 – Wave 8)	Frequency of engagement in visiting: (i) theater, concert or opera, (ii) cinema, (iii) art gallery, exhibition or museum, considering infrequent participation (‘never’, ‘less than once a year’, ‘once or twice a year’) *vs* frequent participation (‘every few months’, ‘about once a month’, ‘twice a month or more’)	Depression
Fushiki, 2012 (22)	Japan	3,583 (1,955 participants completed the questionnaires 3 times during the study period)	65–84	4 groups: Solitary physical activities; Group physical activities; Solitary cultural activities; Group cultural activities	Longitudinal (collected annually from 2007 to 2010 and completion of the questionnaire three times during this period)	Number of cultural activities (in solitary [music appreciation, ceramics, handicrafts, etc.] and in group [cultural club, tea klatch, Japanese chess, etc.]). Yes/no responses. The author also considered the participation in physical activities, this aspect is not taken into account in this review.	Frailty
Keisari, 2021 (15)	Israel	205 (67.3%)	65–92 (*M* = 72.3)	1 group	Cross-sectional	Receptive arts engagement (visiting theater, concerts, dance performance, art galleries, museums, and the cinema) on a single-item scale ranging from 1 (not at all) to 6 (at least once a week) during the year preceding the COVID-19 outbreak	Anxiety for COVID-19
Rapacciuolo, 2016 (23)	Italy	571 (50.6%)	>60 (*M* = 70.1)	1 group	Cross-sectional	Participation and Nonparticipation in Cultural and Social Activities	Perceived psychological well-being; Resilience
Rogers, 2020 (24)	UK	4,575 (52.7%)	52–99 (*M* = 64.7)	1 group (data from ELSA)	Longitudinal (data for a decade: 2004/2005–2014/2015) (Wave 2 –Wave 7)	Frequency of engagement in visiting: (i) theater, concert, or opera, (ii) cinema, (iii) art gallery, exhibition or museum on a six-point Likert scale (from ‘never’ to ‘twice a month or more’) generating an overall frequency of cultural engagement with 4 intensity levels (“never,” “less than once a year,” “once or twice a year,” “every few months or more”)	Frailty
Takeda, 2015 (25)	Japan	16,642 (50.8%)	50–59 (*M*_M_ = 54.7; *M*_F_ = 54.7)	1 group (data from the population-based survey LSMEP)	Longitudinal (six-year panel survey: 2005/2010)	Hobbies or cultural activities in the last year, classifying those who answered affirmatively as “active” and the others as “inactive”	Mental health status
Tymoszuk, 2020 (26)	UK	Cross- sectional: 6,222 (53.5%). Longitudinal (7 years): 3,127 (55.1%).	Cross-sectional: *M* = 65.6 Longitudinal: *M* = 62.5	2 groups (data from ELSA)	Longitudinal (data for a decade: 2004/2005–2014/2015) (Wave 2 –Wave 7)	Frequency of engagement in visiting: (i) the cinema, (ii) art galleries, exhibitions or museums, (iii) the theater, concerts, or the opera on a four-point Likert scale (‘never’, ‘less than once a year’, ‘once or twice a year’, ‘every few months or more’) creating a binary variable (if every few months or more often = frequent engagement, otherwise less frequent)	Loneliness
Tymoszuk, 2020 (27)	UK	2,767 (54%)	*M* = 62.3	1 group (data from ELSA)	Longitudinal (data for a decade: 2004/2005–2014/2015) (Wave 2 - Wave 7)	Frequency of engagement in visiting: (i) the cinema, (ii) art galleries, exhibitions or museums, (iii) the theater, concerts, or the opera (‘never’, ‘less than once a year’, ‘once or twice a year’, ‘every few months’, ‘once a month or more’); subsequently coded as a binary variable ‘non-frequently engaged’ [‘never’, ‘less than once a year’, ‘once or twice a year’] and ‘frequently engaged’ [‘every few months or more’]. Concerning the six waves (scores ranging from 0 to 6): 0 = ‘no or infrequent arts engagement at all waves’, 1 = ‘short-term engagement’ (frequent engagement at one wave only), 2 = ‘repeated engagement’ (frequent engagement at 2–3 waves), ‘sustained engagement’ (frequent engagement at 4–6 waves).	Experienced well-being, Evaluative well-being and Eudaimonic well-being

ELSA, English Longitudinal Study of Aging; LSMEP, Longitudinal Survey of Middle-aged and older Persons.

**Table 2:** Effects and significance of the impact of various cultural activities on the considered variables.

Author, year	Outcome measure	Cultural variable	*n*	Statistical Indicators ± SE / (SE) (95% IC)	Effect and significance
Fancourt, 2018 (20)^a^	Verbal memory (participants had to remember as many words as possible immediately and after a short delay during which they completed other cognitive tests)	Visiting galleries/museums			
		*less than once a year*	954	0.19 ± 0.14 (−0.08–0.47)	*ns*
		*once or twice a year*	767	0.52 ± 0.15 (0.22–0.82)	+***
		*every few months*	491	0.67 ± 0.18 (0.32–1.02)	+***
		*monthly or more*	178	0.74 ± 0.26 (0.23–1.25)	+**
		Theater/concert/opera			
		*less than once a year*	742	0.41 ± 0.16 (0.10–0.72)	+**
		*once or twice a year*	873	0.24 ± 0.16 (−0.07–0.55)	*ns*
		*every few months*	662	0.63 ± 0.17 (0.29–0.97)	+***
		*monthly or more*	270	0.80 ± 0.24 (0.33–1.27)	+***
		Cinema			
		*less than once a year*	845	0.16 ± 0.14 (−0.12–0.44)	*ns*
		*once or twice a year*	643	0.17 ± 0.15 (−0.13–0.48)	*ns*
		*every few months*	585	0.56 ± 0.17 (0.23–0.89)	+***
		*monthly or more*	284	0.25 ± 0.22 (−0.18–0.67)	*ns*
	Semantic fluency (by asking participants to think of as many words of a particular category as possible in less than 1 min)	Visiting galleries/museums			
		*less than once a year*	954	0.26 ± 0.29 (−0.31–0.82)	*ns*
		*once or twice a year*	767	1.02 ± 0.31 (0.41–1.62)	+***
		*every few months*	491	1.75 ± 0.36 (1.04–2.46)	+***
		*monthly or more*	178	1.20 ± 0.50 (0.22–2.19)	+*
		Theater/concert/opera			
		*less than once a year*	742	0.58 ± 0.32 (−0.05–1.20)	*ns*
		*once or twice a year*	873	0.74 ± 0.32 (0.12–1.37)	+*
		*every few months*	662	1.23 ± 0.34 (0.56–1.91)	+***
		*monthly or more*	270	1.41 ± 0.47 (0.50–2.32)	+**
		Cinema			
		*less than once a year*	845	0.66 ± 0.30 (0.07–1.25)	+*
		*once or twice a year*	643	0.65 ± 0.31 (0.04–1.25)	+*
		*every few months*	585	0.76 ± 0.35 (0.06–1.45)	+*
		*monthly or more*	284	0.14 ± 0.39 (−0.63–0.91)	*ns*
Fancourt, 2018 (19)^b^	Dementia (Informant Questionnaire on Cognitive Decline in the older adults - IQCODE)	Visiting art galleries and museums			
		*Never*	1,279	1	
		*Less than once a year*	1,048	0.92 (0.15) (0.67–1.26)	*ns*
		*Once or twice a year*	845	0.76 (0.14) (0.53–1.09)	*ns*
		*Every few months*	739	0.51 (0.13) (0.32–0.83)	+**
Takeda, 2015 (25)^c^	Mental health status (Kessler 6 (K6) scale - Japanese version)	Hobbies or cultural activities			
		Women			
		*Inactive*	2,934	1	
		*By oneself*	1,107	0.74 (0.62–0.90)	+**
		*With others*	4,291	0.76 (0.67–0.87)	+***
		*Both solo and in group*	135	0.55 (0.33–0.93)	+*
		Men			
		*Inactive*	3,392	1	
		*By oneself*	1,350	0.96 (0.81–1.15)	*ns*
		*With others*	3,317	0.83 (0.71–0.97)	+*
		*Both solo and in group*	116	0.85 (0.39–1.86)	*ns*
Keisari, 2021 (15)^d,e^	COVID-19 Anxiety (Generalized Anxiety Disorder - GAD-7)	Receptive arts engagement	205	−5.10 (0.24) (−0.99–0.03) (art) 0.64 (0.24) (0.16–1.12) (moderation effect)	+* +**
Fancourt, 2019 (21)^f^	Depression experienced over 12 years (8-item Center for Epidemiologic Studies Depression Scale - CES-D)	Cultural engagement			
		*Never*	89	1	
		*Less than once a year*	104	0.80 (0.54–1.19)	*ns*
		*Once or twice a year*	174	0.74 (0.51–1.06)	*ns*
		*Every few months*	167	0.68 (0.47–0.99)	+*
		*Once a month or more*	82	0.52 (0.34–0.80)	+**
Fushiki, 2012 (22)^g^	Incident frailty (3-items questionnaire). Incident frailty was defined as being newly institutionalized or bedridden at home because of physical disability or severe cognitive impairment	Solitary cultural activities			
		*No*	39	1	
		*Yes*	15	0.74 (0.40–1.35)	*ns*
		Group cultural activities			
		*No*	46	1	
		*Yes*	8	0.40 (0.19–0.85)	+*
Rogers, 2020 (24)^h^	Frailty incidence (56-items Frailty Index – FI) and frailty trajectory (measured biennially for 10 years - waves 2–7)	Incidence			
		Cultural engagement			
		*Never*	718	1	
		*Less than once a year*	750	1.09 (0.87–1.38)	*ns*
		*Once or twice a year*	1,212	0.92 (0.74–1.14)	*ns*
		*Every few months or more*	1889	0.47(0.63–0.996)	+*
		Trajectory			
		Cultural engagement			
		*Never*	718	1	
		*Less than once a year*	750	−0.0031 (−0.0053 – −0.0010)	+**
		*Once or twice a year*	1,212	−0.0035 (−0.005 - -0.0015)	+**
		*Every few months or more*	1889	−0.0039 (−0.0059 - -0.0019)	+***
Bolwerk, 2014 (16)^i^	Resilience (brief German version of the Resilience Scale - RS-11)	Visual art production	14	Pre-intervention: 60.64 (± 1.71) Post-intervention: 63.50 (± 1.47)	+*
		Cognitive art evaluation	14	Pre-intervention: 62.57 (± 2.32) Post-intervention: 64.79 (± 1.80)	*ns*
Rapacciuolo, 2016 (23)^j^	Perceived psychological well-being (Psychological General Well Being Schedule - PGWB-S); Resilience (Connor-Davidson Resilience Scale CD-RISC2)	Resilience			
		Participating in cultural and social activities	448	6.07 ± 1.58	+***
		Non participating in cultural and social activities	123	5.14 ± 1.83	
		Psychological General Well-Being			
		Participating in cultural and social activities	448	70.53 ± 17.89	+***
		Non participating in cultural and social activities	123	58.95 ± 23.2	
Tymoszuk, 2020 (27)^k^	Experienced well-being – Positive affect (CASP-19); Evaluative well-being – Life satisfaction (Diener’s life satisfaction scale); Eudaimonic well-being - Control and Autonomy (CASP-19); Eudaimonic well-being - Self-realization (CASP-19)	Positive affect			
	
Engagement with cinema			
	
*No or infrequent*	1,572	1	
	
*Short-term*	316	0.80 (0.61–1.03)	*ns*
	
*Repeated*	371	0.99 (0.78–1.26)	*ns*
	
*Sustained*	508	1.14 (0.91–1.43)	*ns*
	
Engagement with galleries/exhibitions/museums			
	
*No or infrequent*	1,695	1	
	
*Short-term*	317	0.98 (0.76–1.28)	*ns*
	
*Repeated*	327	1.05 (0.82–1.35)	*ns*
	
*Sustained*	428	1.25 (0.98–1.61)	*ns*
	
Engagement with theater/concerts/opera			
	
*No or infrequent*	1,409	1	
	
*Short-term*	486	1.17 (0.91–1.51)	*ns*
	
*Repeated*	364	1.10 (0.86–1.41)	*ns*
	
*Sustained*	608	1.42 (1.14–1.77)	+**
	
Life satisfaction			
	
*Engagement with cinema*			
	
*Short-term*	316	0.001 (−0.56–0.56)	*ns*
	
*Repeated*	371	−0.01 (−0.56–0.54)	*ns*
	
*Sustained*	508	−0.24 (−0.74–0.25)	*ns*
	
Engagement with galleries/exhibitions/museums			
	
*Short-term*	317	0.32 (−0.25–0.90)	*ns*
	
*Repeated*	327	0.46 (−0.08–1.00)	*ns*
	
*Sustained*	428	0.76 (0.28–1.25)	+**
	
Engagement with theater/concerts/opera			
	
*Short-term*	486	0.11 (−0.45 – 0.66)	*ns*
	
*Repeated*	364	0.13 (−0.42–0.67)	*ns*
	
*Sustained*	608	0.43 (0.02–0.89)	*ns*
	
Control autonomy			
	
Engagement with cinema			
	
*Short-term*	316	−0.18 (−0.44–0.08)	*ns*
	
*Repeated*	371	0.16 (−0.10–0.43)	*ns*
	
*Sustained*	508	0.06 (−0.17–0.29)	*ns*
	
Engagement with galleries/exhibitions/museums			
	
*Short-term*	317	−0.001 (−0.28–0.27)	*ns*
	
*Repeated*	327	0.28 (0.02–0.54)	+*
	
*Sustained*	428	0.20 (−0.04–0.44)	*ns*
	
Engagement with theater/concerts/opera			
	
*Short-term*	486	0.08 (−0.17–0.33)	*ns*
	
*Repeated*	364	0.33 (0.08–0.58)	+*
	
*Sustained*	608	0.28 (0.05–0.51)	+*
	
Self-realization			
	
Engagement with cinema			
	
*Short-term*	316	0.09 (−0.20–0.39)	*ns*
	
*Repeated*	371	0.09 (−0.17–0.36)	*ns*
	
*Sustained*	508	0.13 (−0.10–0.37)	*ns*
	
Engagement with galleries/exhibitions/museums			
	
*Short-term*	317	0.16 (−0.12–0.44)	*ns*
	
*Repeated*	327	0.31 (0.04–0.58)	+*
	
*Sustained*	428	0.51 (0.27–0.76)	+***
	
Engagement with theater/concerts/opera			
	
*Short-term*	486	0.17 (−0.10 – 0.45)	*ns*
	
*Repeated*	364	0.27 (−0.01–0.54)	*ns*
	
*Sustained*	608	0.30 (0.08–0.53)	+**
Tymoszuk, 2020 (26)^l^	Loneliness (three-item short-form of the Revised UCLA Loneliness Scale)	Engagement with cinema			
		*Never*	937	1	
		*Less than once a year*	776	1.15 (0.87–1.54)	*ns*
		*Once or twice a year*	603	0.91 (0.66–1.27)	*ns*
		*Every few months or more*	811	0.96 (0.78–1.41)	*ns*
		Engagement with art galleries and museums			
		*Never*	908	1	
		*Less than once a year*	883	0.86 (0.65–1.13)	*ns*
		*Once or twice a year*	716	0.74 (0.54–1.01)	*ns*
		*Every few months or more*	620	0.68 (0.48–0.95)	+*
		Engagement with theater/concert/opera			
		*Never*	776	1	
		*Less than once a year*	699	0.75 (0.55–1.02)	*ns*
		*Once or twice a year*	807	0.69 (0.50–0.95)	+*
		*Every few months or more*	855	0.84 (0.61–1.15)	*ns*

***p ≤ 0.001; **p ≤ 0.01; *p ≤ 0.05. ^a^Pre- Post- Model 4 (Ordinary Least Square Regression - β): adjusted for baseline cognition, sex, age, marital status, ethnicity, educational attainment, employment status, occupational classification, wealth, self-reported health, eyesight, hearing, depression, social network, civic engagement, whether participants had a hobby, whether participants used the internet and whether participants read a daily newspaper. ^b^Model 4 (Poisson regression - Incidence Rate Ratio (IRR) of dementia incidence and 95% Cis): adjusted for gender, age, marital status, educational attainment, employment, wealth and occupational classification, eyesight, hearing, depression and existing cardiovascular health conditions, community engagement. ^c^Model (Multiple logistic regression models - AOR): adjusted for demographic and socioeconomic status, physical health condition, chronic diseases and mental health status at the baseline. ^d^Cross-sectional - Multiple hierarchical regression analysis (moderation effect of culture between resilience and anxiety) (B); ^e^Multiple hierarchical regression model (step 2: Resilience *p* < 0.00 and Art *p* < 0.04; step 3: Resilience × Art *p* = 0.01). ^f^Model 3 (Logistic regression analyses - OR): adjusted for age, gender, SES & baseline depression. ^g^Cox Proportional Hazard Models (Hazard ratios - HRs and 95% Cis) for frailty associated with participation in hobby activities. HRs adjusted for sex, age, self-perceived health status, cerebrovascular disease, smoking and drinking habits and body mass index. ^h^Incidence and trajectory: Model 2 (Competing risk regression model Sub-hazard ration – SHR and 95% CI), adjusted for covariates and baseline frailty (non-frail vs pre-frail). ^i^Wilcoxon signed-rank test (Median and standard error of the mean – SEM). ^j^Cross-sectional - Student’s t-test between “Participant” and “Non-Participant” groups; mean and ± standard deviation was reported. ^k^Positive affect: Logistic regression model was reported Odds Ratio (OR), 95%; for Life satisfaction, Control autonomy, Self-realization: Linear Regression Model was reported B and 95% CI. All model was adjusted for: Wave 2 well-being score, gender, age, ethnicity, coupled relation status, highest educational attainment, employment status, and net non-pension wealth, eyesight and hearing problem, experiences of pain and chronic illness, social isolation index, and civic activities. ^l^Model 5 (Logistic regression analyses – OR): univariate + demographic factors (gender, age, ethnicity, highest educational attainment, employment status, and net non-pension wealth + health factors) (eyesight and hearing problems, experiences of pain and long-standing illness status) + social factors (social contact, romantic relationship status, and engagement with community activities) + baseline loneliness score.

Several studies used data from national databases (*n* = 6). All studies used a quantitative methodology. Concerning the research designs, most of the studies were longitudinal (*n* = 7, one of which is retrospective), since cross-sectional (*n* = 2), a follow-up survey and an RCT. The time elapsed between the initial data collection and subsequent data collection in longitudinal studies typically ranged from 6 to 10 years. Sample sizes varied considerably, from 28 participants (RCTs) to large national surveys with 16,642 participants. The majority of the studies were conducted in the United Kingdom (*n* = 6), with Japan (*n* = 2), Italy (*n* = 1), Israel (*n* = 1), and Germany (*n* = 1) also represented. The age range of participants spanned from 50 to 99 years, with a balanced gender distribution.

The psychological and social health outcomes varied significantly. The most prominent variables examined were resilience (*n* = 2), well-being (*n* = 2) and frailty (*n* = 2), followed by depression (*n* = 1), anxiety (*n* = 1), mental health (*n* = 1), dementia (*n* = 1), cognitive functions (memory and semantic fluency; *n* = 1) and loneliness (*n* = 1). Except for the RCT, which introduced specific cultural activities, the remaining studies focused on regular, ongoing cultural participation.

### 3.2 Quality assessment

9 studies displayed a high methodological quality, whereas 2 studies received moderate quality ratings due to (a) a non-clear description of the criteria for inclusion in the sample as well as for the study subjects and the setting (*n* = 1), and (b) the non-identification of confounding factors (*n* = 1). The authors of 7 studies utilized data from national databases, which did not permit a clear *a priori* specification of inclusion criteria beyond age. Nevertheless, they expanded the survey to encompass large samples and provided adequate descriptions.

### 3.3 Health and well-being outcome

The order of the discussed outcome aligns with the principles of the biopsychosocial model: first, “Cognitive Functioning” addresses the fundamental aspects of brain biology; then, “Dementia” is explored due to its involvement in cognitive processes; “Mental Health” encompasses a spectrum of psychological aspects; “Frailty” acts as a crucial connector, spanning individual and societal domains; “Resilience” acknowledged as both personal and social resource; “Well-being” is examined for its multifaceted determinants, including social influences; finally, “Social Relationships” for their direct involvement in social interaction. The decision to separate the discussion by theme stems from the diverse methods and variables considered in the selected studies.

#### 3.3.1 Cognitive functioning

Fancourt and Steptoe (20) found that cultural participation in general has a positive impact in terms of cognitive conservation, verbal memory and semantic fluency, especially if adequately sustained (at least a couple of times a year), regardless of baseline cognitive status and other variables (e.g., demographics, health, etc.). Particularly, a dose–response relationship emerges, indicating that a higher frequency of visits to galleries or museums, as well as theaters, concerts, or opera, had a greater effect on cognition with a protective effect. The results regarding the association between going to the cinema and cognitive function become less clear and consistent when other control factors are considered and corrected for multiple comparisons. On the whole, the reported results show that the activities were protective regardless of the median level of baseline cognition.

#### 3.3.2 Dementia

Visiting museums could be a promising psychosocial activity to support dementia prevention, especially if sustained over time (19) The reported results show that such activity is associated with a lower incidence rate of dementia over a 10-year follow-up period in individuals aged over 50. The incidence rate of dementia is lower among individuals who regularly attend museums compared to those who do not attend museums. Particularly, the overall incidence rate was 5.42 (95% CI 4.78–6.17) per 1,000 person-years; the incidence rate resulted higher than average for non-participants (Δ = 4.05), slightly lower than average for sporadic participants (less than once a year: Δ = −1.46; once or twice a year: Δ = −1.69), and even lower for those who visited galleries and museums frequently (Δ = −3.27) (19). Taken into account the demographic differences, the association between cultural participation and a dementia remained significant only for those who visited museums every few months or more.

#### 3.3.3 Mental and psychological health

Participation in recreational activities (hobbies/cultural activities) showed a positive association with mental health after a five-year follow-up (25). Participating in activities with others has a positive impact on mental health, and this effect is particularly marked when compared to those who do not engage in any social activities. These differences are also notable between genders. Whereas this association was observed among men in a larger sample, women showed a positive relationship with mental health regardless of the mode of participation in group activities (25). Keisari et al. (15) found that receptive artistic engagement moderated the relationship between resilience, conceptualized as an individual’s ability to effectively cope with and adapt to the challenges and difficulties brought about by the coronavirus pandemic and COVID-19 anxiety. Specifically, the significant interaction between resilience and receptive arts engagement accounted for an additional 3% of the variance in anxiety symptoms. Furthermore, the authors found that pre-pandemic cultural participation had a buffering effect against COVID-19 anxiety; conversely, individuals with low artistic involvement reported higher levels of anxiety. Fancourt and Tymoszuk (21) confirmed that a regular and sustained cultural engagement (at least every few months) represents an important risk reducing factor for the development of depression in older age. A clear dose–response relationship emerges, indicating that higher frequency of participation is associated with a reduced risk. Those who rarely or never participate (once or twice a year) showed an incidence rate of depression above the average, whereas higher participation frequencies were linked to rates below the average.

#### 3.3.4 Frailty

Rogers and Fancourt (24) found a dose–response relationship between cultural participation and both the incidence and progression of frailty. Regarding the incidence, the authors found a subhazard ratio of 0.92 CI [0.85–0.98] between frequency of cultural engagement and incidence frailty. Moreover, the risk of frailty at the age of 80 is 1.3 times higher for those who do not engage in cultural activities, independent of confounding factors such as demographics, socioeconomic status, and social factors. These findings corroborate those of a prior study by Fushiki and colleagues (22), which indicated that individuals who participated - in their life - in at least one or more cultural or physical group activities after adjustment exhibited a lower incidence of frailty compared to those who engaged in such activities alone. Furthermore, when comparing cultural and physical activities (solo or in groups), individuals participating in one or more cultural activities demonstrated a lower incidence of frailty.

#### 3.3.5 Resilience

Bolwerk and coll (16) showed that the cultural engagement can increase resilience, conceptualized as a protective personality trait enabling individuals to mitigate the negative impacts of stress and facilitating successful and healthy functioning even amidst challenging life circumstances. Although the effects were greater and statistically significant only in the “Visual art production” group (the resilience level increased by 2.86 points between pre- and post-intervention), a non-significant improvement also emerged in the “Cognitive art evaluation” group (+2.22). These results are also confirmed at the biological level: using fMRI, they observed that participants engaged in visual art production, compared to the assessment of art, showed greater spatial improvement in functional connectivity in different brain areas (mostly between the parietal and frontal cortices) over time, and that this was related to psychological resilience. Rapacciuolo and coll (23) showed that those who participate in cultural and social activities (both women and men) have higher levels of resilience, define as successful stress-coping ability, compared to non-participants (+ 0.93).

#### 3.3.6 Well-being

As previously mentioned, Rapacciuolo and coll (23) showed an association between participation in cultural activities (mostly for women) and psychological well-being: who participate in cultural and social activities have higher levels of well-being compared to non-participants (+ 11.58). Participation in social and cultural activities, along with interventions aimed at fostering positive emotions, could be crucial in combating social isolation and its adverse effects on health. Additionally, as suggested by the authors, these activities may contribute to promoting healthier lifestyles, such as improving nutrition. Tymoszuk and coll (27) showed that sustained (once a month or more) cultural participation has a positive impact on various forms of well-being. Considering experienced well-being, sustained engagement with the theater/concert/opera compared with no or infrequent engagement showed a positive effect (OR = 1.4, 95% CI 1.14–1.77, *p* = 0.02). Moreover, about evaluative well-being, sustained engagement with gallery/museum compared with no or infrequent engagement was associated with higher life satisfaction (B = 0.76, 95% CI: 0.28, 1.25, *p* = 0.002). In addition, regarding eudaimonic well-being, sustained engagement with galleries/exhibitions/museums was associated with higher self-realization if compared to no or infrequent engagement (B = 0.51, 95% CI: 0.27, 0.76, *p* < 0.001). Finally, considering again eudaimonic dimension, sustained engagement with the theater/concerts/opera respect to no or infrequent engagement was related with higher control/autonomy (B = 0.28, 95% CI: 0.05, 0.51, *p* = 0.018) and self-realization (B = 0.30, 95% CI: 0.08, 0.53, *p* = 0.008). No associations were found for engagement with screen-based performances (cinema attendance), in contrast to studies that have demonstrated its beneficial effects but in line with other studies that have identified positive associations between time spent in front of screens (TV) and depressive symptoms, sedentary behavior, and other factors.

#### 3.3.7 Social relationships

Tymoszuk and coll (26) used the second wave of ELSA for the cross-sectional analyses and data from the seventh wave (a decade later) for the longitudinal analyses. The cross-sectional results showed that: engaging with cinema every few months or more often, compared with never, was associated with 26% lower odds of loneliness, visiting galleries/exhibitions/museums every few months or more often and once or twice a year had, respectively, 26 and 22% lower odds of loneliness compared with those who reported no engagement. Participants who reported attending theater, concerts, or opera every few months or more frequently, as well as those attending once or twice a year, exhibited 33 and 23% lower odds of experiencing loneliness, respectively, compared to those who reported no engagement in such activities. However, longitudinal analysis revealed no association between the frequency of cinema attendance and the likelihood of experiencing loneliness, even after adjusting for covariates. Engaging with galleries, exhibitions, and museums every few months or more often, compared to never, was associated with a 32% reduction in the odds of experiencing loneliness at wave 7. Similarly, engaging once or twice a year was linked to a 26% decrease in the likelihood of reporting loneliness at wave 7 after adjusting for covariates. In the fully adjusted model, participating in theater, concerts, or opera once or twice a year, compared to never, was associated with a 31% decrease in the odds of experiencing loneliness at wave 7. The longitudinal analytical sample exhibited skewness toward participants who were female, younger, employed, more educated, in good health, in coupled relationships, reported higher levels of social, community, and arts engagement, and were less likely to be lonely at wave 2. In general, the participation in receptive artistic activities is negatively associated with the risk of loneliness especially for attending museums/galleries/exhibitions compared to theater/concerts/opera and visits to the cinema. This effect emerged regardless of the baseline loneliness level and different confounding variables (i.e., demographic, socioeconomic, health and social factors).

## 4 Discussion

The results of this systematic review suggest that cultural engagement may be effective in maintaining and enhancing health and well-being of middle-aged and older populations. Regarding our first research question, the evidence suggests that cultural activities have a positive impact on various dimensions of well-being. Visiting museums, galleries, and exhibitions provides positive cognitive stimulation, reducing the risk of cognitive decline or the development of dementia (19). Indeed, there is a relationship between the frequency of museum visits and the incidence rate of dementia, with a lower rate among those who participate more in this activity, and these results remain significant even after accounting for demographic and health variables (19). Moreover, art exhibitions as well as live performances have a positive impact on memory and semantic fluency, reducing decline in cognitive function compared to non-participation (20). Longitudinal associations spanning a decade were observed independent of initial indications of cognitive decline, indicating that cultural engagement may yield benefits also for individuals experiencing cognitive impairment (20). Overall, the results concerning cognitive dimension support the assumption that «cultural engagement […] contributes to cognitive reserve: the resilience of our brains as we age» (4) (p. 24). According to Stern (28), the cognitive reserve against brain damage allows people to deal with cognitive decline; this hypothesis supports the idea that the reserve factors derive from different cognitive dimensions, including education level and intelligence (*cf.* (29)), and participation in specific activities (e.g., cultural activities), which act as protective factors against brain disease (28). The studies reveal intriguing benefits of cultural engagement on psychological resilience at the cerebral level as well: engaging in visual arts has been found to enhance the interaction between various brain regions, thereby improving the ability to endure or cope with challenging situations (16). Furthermore, a high degree of involvement in the arts can potentially act as a protective barrier against specific emotional responses, effectively serving as a moderator between resilience and COVID-19-related anxiety, demonstrating its efficacy as a coping strategy (15). Especially for individuals with low involvement in receptive arts, increased resilience significantly reduced anxiety symptoms; therefore, both context and personal resources influence how resilience and engagement in the arts combine to affect anxiety. Receptive arts engagement has been shown to enhance psychological resources in older age, thereby reducing the risk of developing mental health problems (25). The results suggest that sailing in shared experiences can yield significant benefits for mental health. Overall, socialization and interaction with others represent an added value. Notably, compelling associations have emerged between consistent participation in cultural activities and subjective dimensions of well-being, encompassing both subjective and psychological aspects (23). Additionally, it serves as a protective factor for older individuals, mitigating the risk of mental illnesses such as depression (21). In the realm of cultural engagement, older adults find a sovereign refuge against depression, woven with threads of social interaction, mental creativity, and cognitive stimulation. The advantages of arts engagement in older age extend to frailty trajectories, effectively reducing the incidence and progression of physiological decline and providing protection against vulnerability to adverse health outcomes (24). Notably, this study represents the initial evidence supporting the potential significance of cultural engagement in older age in reducing both the risk of developing frailty and the trajectory of its progression (24). Finally, at a social level, sustained engagement with museums, galleries, and exhibitions protects against loneliness. Several studies have shown that life events which tend to occur in older age can increase the risk of social isolation and feelings of loneliness (30). This is a very important effect since loneliness negatively affects psychophysical well-being, exacerbating cognitive decline and progression of dementia, increasing the risk of premature mortality (31). Whereas some studies tend to attribute the benefits of cultural engagement, for example, to reducing social isolation, further analysis reveals the relevance of other aspects, such as pleasure experiences and emotional expression (24). Therefore, social benefit is not the sole important factor contributing to the positive health effects. A more critical analysis of this literature might shed further light on this. In a kind of melody of interconnection, the presence of others during recreational activities could play a pivotal role in promoting health, suggesting an interconnectedness between social engagement and positive health outcomes in the realm of cultural activities (25).

In summary, according to the recent scoping review of Fancourt and Finn (4), this systematic review highlights the potential of cultural participation in promoting healthy aging. In accordance with the WHO Global Strategy and Action Plan on Aging and Health, healthy aging is “the process of developing and maintaining the functional ability that enables well-being in older age” (32). These findings emphasize that a regular and sustained cultural engagement, especially in group, can enhance or maintain the well-being while also serving as a preventive measure against potential psychophysical and social disorders and challenges. However, some limitations were observed. In certain studies, various leisure activities and cultural activities were grouped together as a single variable, making it difficult to isolate the impact of specific cultural participation forms. An issue also arises due to the self-reported and retrospective measurement of cultural involvement. Consequently, the data may not always be accurate and may not fully capture the true value of participation in such activities. Numerous studies, especially those utilizing ELSA data, did not thoroughly explore active participation by separating the different activities. In some cases, the assessment of this multifaceted activity was simplified to a single item, despite the diverse effects demonstrated in the reviewed literature across various forms of participation. Furthermore, due to the observational nature of the data (with only one randomized controlled trial included in this review), caution is required when inferring causal relationships between cultural engagement and the various outcomes. The primary findings suggest bidirectional associations, indicating susceptibility to reverse causality bias. Indeed, it is possible that mostly healthy people tend to participate in such activities.

To the best of our knowledge, this is the first systematic review that specifically focuses on the healthy population aged over 40, exclusively considering the psychophysical and social effects of cultural participation. Moreover, our study did not limit the selection of research to randomized controlled trials (RCTs), but also included longitudinal studies based on national databases and cross-sectional studies. We conducted the review by searching various electronic databases with no restrictions on publication dates. The independent analysis conducted by two team members, focusing of both study quality and results, further strengthens the credibility of our review. The studies considered in our analysis were conducted in various geographic regions, not limited to Western countries, thereby providing cross-cultural validation of the value of cultural participation.

Obviously, conducting a meta-analysis could provide empirical evidence regarding the value of cultural participation. However, the variations in methods used to measure this type of activity, along with the diverse range of outcomes considered, hinder the feasibility of such an approach. Additionally, our selection was limited to studies with samples aged over 40, but it could be of interest to explore broader age groups in future research to uncover potential differences that may arise at various stages of life.

In light of the limitations observed in the current literature, there are some future topics to investigate. First, efforts should be made to reduce heterogeneity. This can be achieved by developing a more standardized measure and the definition of culture and cultural participation. Additionally, it is crucial to distinguish between different forms of cultural engagement, as this review has shown that some activities are less effective than others (e.g., cinema attendance). Furthermore, future studies should aim to minimize reliance on self-reported measures of participation and instead utilize standardized measures. Lastly, researchers should consider the aspect of active cultural participation, which involves individuals in the creation of artistic works. This transformation shifts the passive viewer into an active participant or artist, potentially yielding unique insights into the relationship between culture and well-being. A fundamental distinction arises between active participation, where individuals directly engage in the creative process, and receptive engagement (i.e., attending arts events or listening to music). These distinctions result in significant variability that need for consideration in future studies aimed at advancing our understanding of the complex relationship between culture and health (11, 12). To address the problem of revers causality, future studies should consider adopting experimental design, RCTs and consistently include a control group or condition.

## 5 Conclusion

Our results are encouraging. The primary finding from this systematic review suggests that sustained cultural participation appear to have a positive impact on various dimensions—biopsychosocial—of health and well-being, highlighting the importance of culture for middle-aged and older populations. Those who engage in cultural activities show an improvement in terms of well-being, or at the very least, a maintenance of their health status. Further research, particularly RCTs with control conditions, is needed to gain a deeper understanding of the mechanisms by which cultural participation influences health and well-being outcomes and to develop effective intervention strategies. These studies should employ robust multidimensional measures and also explore potential moderators and mediators, ultimately enhancing the development of future interventions. These findings present a valuable opportunity for multidisciplinary collaboration between healthcare, sociocultural sectors, and arts-related systems and policies.

## Author contributions

EV: Conceptualization, Data curation, Formal analysis, Investigation, Methodology, Project administration, Writing – original draft, Writing – review & editing. MM: Data curation, Formal analysis, Investigation, Writing – original draft, Writing – review & editing. DC: Conceptualization, Data curation, Investigation, Writing – review & editing. MV: Conceptualization, Data curation, Investigation, Writing – review & editing. DA: Investigation, Writing – review & editing. FF: Conceptualization, Funding acquisition, Supervision, Writing – review & editing.
