# Corrigendum: The effects of cultural engagement on health and well-being: a systematic review

**DOI:** 10.3389/fpubh.2024.1508337

**Published:** 2024-10-21

**Authors:** Erica Viola, Marco Martorana, Daniele Ceriotti, Marta De Vito, Damiano De Ambrosi, Fabrizio Faggiano

**Affiliations:** ^1^Department of Sustainable Development and Ecological Transition, University of Eastern Piedmont, Vercelli, Italy; ^2^Department of Statistics, Computer Science, and Applications “Giuseppe Parenti” (DiSIA), University of Florence, Florence, Tuscany, Italy; ^3^Department of Translational Medicine, University of Eastern Piedmont, Novara, Piedmont, Italy

**Keywords:** cultural engagement, leisure activities, health, well-being, quality of life

In the published article, there was an error in the Funding statement. The details for project AGE-IT were missing. The correct Funding statement appears below.

