# Supplementary material for: The effects of cultural engagement on health and well-being: a systematic review
Source: Front Public Health. 2024 Jul 10;12:1369066. doi: 10.3389/fpubh.2024.1369066 (PMC11266038; doi:10.3389/fpubh.2024.1369066)
Supplement: Supplementary file 1 [file Table_1.DOCX]

**The effects of cultural engagement on health and wellbeing: a systematic review**

**Supplementary material**

**Table A1.** summarising the results of the Critical Appraisal Checklist for Analytical Cross-Sectional Studies (Moola et al., 2020).

|  | Bolwerk, 2014 | Fancourt, 2018a | Fancourt, 2018b | Fancourt, 2019 | Fushiki, 2012 | Keisari, 2021 | Rapacciuolo et al., 2016 | Rogers, 2018 | Takeda, 2015 | Tymoszuk, 2020a | Tymoszuk, 2020b |
| --- | --- | --- | --- | --- | --- | --- | --- | --- | --- | --- | --- |
| Were the criteria for inclusion in the sample clearly defined? | Y | NA | NA | NA | Y | N | N | NA | NA | NA | NA |
| Were the study subjects and the setting described in detail? | Y | Y | Y | Y | Y | Y | N | Y | Y | Y | Y |
| Was the exposure measured in a valid and reliable way? | Y | Y | Y | Y | Y | Y | Y | Y | Y | Y | Y |
| Were objective, standard criteria used for measurement of the condition? | Y | Y | Y | Y | Y | Y | Y | Y | Y | Y | Y |
| Were confounding factors identified? | N | Y | Y | Y | Y | Y | Y | Y | Y | Y | Y |
| Were strategies to deal with confounding factors stated? | NA | Y | Y | Y | U | Y | Y | Y | Y | Y | Y |
| Were the outcomes measured in a valid and reliable way? | Y | Y | Y | Y | Y | Y | Y | Y | Y | Y | Y |
| Was appropriate statistical analysis used? | Y | Y | Y | Y | Y | Y | Y | Y | Y | Y | Y |
| **Overall**  **Methodological**  **Quality** | **M** | **H** | **H** | **H** | **H** | **H** | **M** | **H** | **H** | **H** | **H** |

*Note*: Y=Yes; N=No; U=Unclear; NA=Not applicable; L=Low (<5); M=Moderate (<7); H=High (≤8).
